# Ionoprinted Multi-Responsive Hydrogel Actuators

**DOI:** 10.3390/mi7060098

**Published:** 2016-05-26

**Authors:** Daniel Morales, Igor Podolsky, Russell W. Mailen, Timothy Shay, Michael D. Dickey, Orlin D. Velev

**Affiliations:** Department of Chemical and Biomolecular Engineering, North Carolina State University, Raleigh, NC 27695, USA; dhmorale@ncsu.edu (D.M.); igorpodolsky@umail.ucsb.edu (I.P.); rwmailen@ncsu.edu (R.W.M.); twshay@ncsu.edu (T.S.)

**Keywords:** hydrogel actuators, soft robotics, stimuli responsive systems, ionoprinting, pNIPAAm

## Abstract

We report multi-responsive and double-folding bilayer hydrogel sheet actuators, whose directional bending response is tuned by modulating the solvent quality and temperature and where locally crosslinked regions, induced by ionoprinting, enable the actuators to invert their bending axis. The sheets are made multi-responsive by combining two stimuli responsive gels that incur opposing and complementary swelling and shrinking responses to the same stimulus. The lower critical solution temperature (LCST) can be tuned to specific temperatures depending on the EtOH concentration, enabling the actuators to change direction isothermally. Higher EtOH concentrations cause upper critical solution temperature (UCST) behavior in the poly(*N*-isopropylacrylamide) (pNIPAAm) gel networks, which can induce an amplifying effect during bilayer bending. External ionoprints reliably and repeatedly invert the gel bilayer bending axis between water and EtOH. Placing the ionoprint at the gel/gel interface can lead to opposite shape conformations, but with no clear trend in the bending behavior. We hypothesize that this is due to the ionoprint passing through the neutral axis of the bilayer during shrinking in hot water. Finally, we demonstrate the ability of the actuators to achieve shapes unique to the specific external conditions towards developing more responsive and adaptive soft actuator devices.

## 1. Introduction

There has been intense interest and progress in engineering soft, shape-transforming materials, such as hydrogels, which can mimic the sensing and response mechanisms found in nature. Hydrogels can undergo large volume changes in response to a multitude of external stimuli, which alter the polymer–solvent interactions [1,2,3,4,5]. The stimulus-induced volume changes can be harnessed to create actuators by bonding together two or more materials that swell differently in response to the same stimuli. Examples include bilayer gel systems [6,7,8,9,10,11,12,13,14,15,16], particle or interpenetrating polymer hydrogel composites [17,18,19,20] and gels with regions of varying crosslink density both through the gel depth or in-plane [21,22,23,24,25,26,27]. The differential swelling leads to a buildup of internal stresses resulting in reversible, 3D shape transformations of 2D sheets. Such adaptive materials could be applied to a broad range of applications including biomaterials [28,29], soft robotics [30,31], drug delivery [32,33], microfluidics [34] and sensing [35,36]. Gels have several challenges as actuators including slow response times [37,38] and weak mechanical properties [39]. Furthermore, previously explored actuation mechanisms generally result in an “on” or “off” state, determined by the magnitude of the applied stimulus. There is interest in developing synthetic systems that reconfigure into 3D structures that are uniquely and proportionally responsive to a specific set of external conditions, towards developing “intelligent” soft materials.

A number of strategies exist to create multi-responsive gel actuators by incorporating multiple penetrating polymer networks sensitive to different stimuli into one gel composite [18,27,40], incorporating bi-axial stress with crosslinking gradients [41] or by incorporating modular gel building blocks into 3D geometries [42]. These gel systems have large response times (~min–h) and require multiple photolithographic processing steps.

We sought to explore the shape changes made possible by incorporating two active gel layers combined with an ionic crosslinking technique, ionoprinting, developed previously in our research [21]. Here, we have introduced multi-responsive gel bilayer sheets by combining thermoresponsive poly(*N*-isopropylacrylamide) (pNIPAAm) with superabsorbent poly(sodium acrylate) (pNaAc) gels. The actuators can be created rapidly, with simple bench top equipment and are functionalized with reversible, locally crosslinked regions. The bending direction can be tuned independently by changing the solvent quality or by changing the external temperature. We achieve this by making use of the LCST (lower critical solution temperature) and UCST (upper critical solution temperature) transitions that pNIPAAm can incur at certain concentrations of the appropriate co-nonsolvent [43]. Various groups have utilized the LCST phase transition of pNIPAAm gels to develop a variety of actuators, which can achieve complex shapes by shrinking below the critical temperature [4,10,14,17,19,20,22,44]. The UCST phase change has been relatively unexplored in the context of hydrogel actuation and can impart additional functionality to existing pNIPAAm actuation mechanisms. The bilayer bending axis is determined by the location of stiff, highly crosslinked ionoprinted regions [21]. This double curvature effect has been discussed previously as means of achieving complex hydrogel shape transformation [40] and demonstrated experimentally with PDMS bilayer sheets [45]. Ionoprinting allows for making reprogrammable bilayer sheets due to the ability to erase the ionic crosslinks with a chelating agent. We combine the strategies of bi-directional bilayer actuation with stiff regions of ionoprinted crosslinking to direct the final 3D shape transformation.

In this work, we demonstrated three modes of programmable actuation, which can be combined to produce reconfigurable 3D hydrogel structures. First, we made and characterized hydrogel bilayers whose bending direction can be tuned by manipulating the pairwise interactions between polymer and solvent by temperature *T*, and solvent quality, characterized by the ethanol concentration, *x*_2_. Second, we controlled the bending axis of these sheets utilizing locally, ionically crosslinked regions made by ionoprinting. Third, we show that the bending axis of the gel bilayer system can be inverted by changing the location of the ionoprint along the sheet cross section. Finally, we demonstrate the use of these simple fabrication techniques to produce gel actuators that transform into unique 3D shapes with a rapid response time.

## 2. Experimental Section

### 2.1. Materials

*N*-isopropylacrylamide (NIPAAm) monomer, anhydrous acrylic acid 99% (AAc, Sigma Aldrich, St. Louis, MO, USA) monomer, *N*,*N*′-methylenebis(acrylamide) (BAAm, Sigma Aldrich) crosslinker, ammonium persulfate (APS, Sigma Aldrich), *N*,*N*,*N*′*N*′-tetramethylethylenediamine 99.5% (TEMED, Sigma Aldrich), Brilliant Green Dye (Sigma Aldrich), poly(ethyleneimine) 50% (*w*/*v*) Mn = 60,000 (PEI, Sigma Aldrich), *N*-(3-Dimethylaminopropyl)-*N*′-ethylcarbodiimide hydrochloride (EDC, Sigma Aldrich) and sodium hydroxide (Acros Chemical, Geel, Belgium) were used as received. The gels were prepared and equilibrated in Milli-Q deionized water (18.2 MΩ·cm).

### 2.2. Hydrogel Polymerization

All hydrogels were prepared by thermal, free-radical polymerization in aqueous solution using BAAm as crosslinker. APS and TEMED were used as the initiator and accelerator, respectively. For isopropylacrylamide (NIPAAm) hydrogels, the overall monomer concentration was fixed at 2.3 M and the crosslinker ratio (mole ratio of divinyl to vinyl monomers) was fixed at 1:25. The NIPAAm precursor solution contained 1.25 wt % of branched PEI chains to impart the pNIPAAm gel with cationic groups [6,46]. This provided sufficient amounts of cationic primary amine groups to interact with the anionic carboxylic groups of the pNaAc gel without affecting the thermo responsive volume change of the pNIPAAm. The monomers were dissolved along with 143 µL of a 10 wt % APS solution in 3.5 mL of water. After addition of 25 µL of TEMED, the monomer solution was injected between clean glass slides separated by 1 mm thick silicon spacers. The gel sheets were left overnight to polymerize and subsequently placed in an excess of Milli-Q water to equilibrate before experimentation.

For sodium acrylate (NaAc) hydrogels, the overall monomer concentration was fixed at 5 M and the crosslinker ratio (mole ratio of divinyl to vinyl monomers) was fixed at 1:150. Sodium acrylate was prepared *in situ* by adding equimolar amounts of acrylic acid and sodium hydroxide to the precursor mixture. The monomers were dissolved along with 67 µL of a 10 wt % APS solution in 5 mL of water. After addition of 25 µL of TEMED, the monomer solution was injected between clean glass slides separated by 100 µm thick Teflon tape. The gel sheets were placed in a 70 °C oven for 3 h to polymerize and subsequently placed in an excess of Milli-Q water to equilibrate before experimentation. The pNaAc gel sheets for tensile extension tests (see Section 2.4) were formed by injecting the mixture between glass slides separated by 1 mm silicone spacers.

### 2.3. Bilayer Fabrication

The gels were electro-adhered by placing sheets in contact between two fluorine-doped tin oxide (FTO) glass electrodes. Directionally applied electric fields (5 V/mm, 30 s) promoted attractive electrostatic interactions between the carboxylic and amine groups at the interface between the oppositely charged polymer networks. After adhesion, submerging the gel bilayers in 10 mM EDC solution for 4 h promoted covalent bond formation between the carboxylic and primary amine groups at the interface between the gels. Finally, the gel bilayers were equilibrated in pure water.

The ionoprints were created by contacting the pNaAc gel with 0.5 mm diameter copper wire and the pNIPAAm with an FTO electrode. Oxidative bias applied to the copper wire with an electric field of 5 V/mm for 10 s created localized regions of high ionic crosslinking. External ionoprinting was performed after the bilayers were equilibrated in pure water. Internal ionoprinting was conducted on individual pNaAc sheets before electro-adhesion of the pNIPAAm sheet.

### 2.4. Mechanical Analysis

Hydrated hydrogel samples were cut in a dog-bone shape according to ASTM D638-10 Type V (gage length = 7.62 mm, neck width = 3.18 mm) [47] with an approximate thickness of 3 mm. Gel samples were subsequently equilibrated in pure water before experimentation. The extensometer elongated the samples at a constant displacement rate of 3 mm/min. The slope of the stress/strain curve within the region of 10% strain was used to obtain the Young’s modulus for the various pNIPAAm and pNaAc gels. Each tensile extension experiment was conducted at least five times and the errors bars were calculated using the data standard deviation.

### 2.5. Bending Curvature Analysis

The radius of curvature as a function of temperature was measured digitally by image analysis using ImageJ software package (Bethesda, Rockville, MD, USA) [48].

## 3. Results

### 3.1. Strategy for Bilayer Design and Bending Direction Control

**Figure 1:**
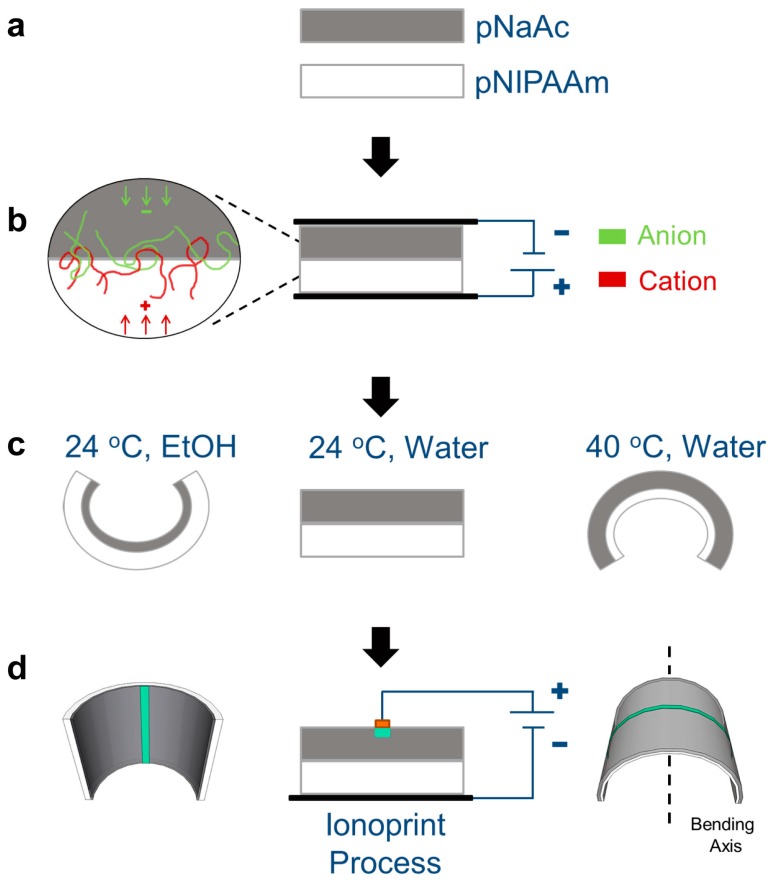
Schematics of the design and of the bending behavior of a hydrogel bilayer. The thermo-responsive gel layer (pNIPAAm) is shown in white and the superabsorbent gel layer (pNaAc) is shown in grey. (**a**) The two layers are allowed to equilibrate in DI water to reach complete swelling at room temperature. (**b**) An applied electric field (5 V/mm) promotes electrostatic polyion complexation at the interface of the two gels. The green chains represent the anionic carboxylic chains of the pNaAc gel and the red chains represent the cationic primary amine group chains of the PEI polymer. (**c**) After adhesion, the bilayer is equilibrated in in a 10 mM EDC solution. The bilayer is flat in room temperature water, and bends toward the pNIPAAm in warm water and toward the pNaAc layer in EtOH. (**d**) By applying an oxidative potential to a copper wire, Cu^2+^ ions complex locally within the anionic hydrogel creating a rigid, highly crosslinked region. In EtOH, the ionoprint is on the interior of the curvature and parallel to the bending axis. In warm water, the ionoprint is on the exterior of the sample and orthogonal to the bending axis.

After polymerization, the hydrogel sheets were immersed in excess DI water to equilibrate to a fully swollen state at room temperature. Equilibrating sheets of pNIPAAm and pNaAc before adhesion resulted in flat bilayers when submerged in room temperature water (Figure 1a). We utilized an electrophoresis driven adhesion technique, reported previously, to form a permanently bound interface [6,46]. Briefly, an electric field applied to a cationic gel at the anode and an anionic gel at the cathode promotes interaction of the ionic groups at the gel/gel interface. Strong polyion complexation due to the high density of charge groups along the pNaAc backbone and physically entangled PEI polymer leads to temporary adhesion [6,46] (Figure 1b). However, these electrostatic interactions can be screened in the presence of electrolytes, leading to separation. We promoted covalent bonding at the interface by immersing the adhered gel sheets in *N*-(3-dimethylaminopropyl)-*N*′-ethylcarbodiimide hydrochloride (EDC) solution [46]. This reaction provides stable, irreversible adhesion at the interface.

Before ionoprinting, the bilayer has three programmable modes: flat, curved toward pNIPAAm, and curved toward pNaAc (Figure 1c). Bonding the two different gel networks enables bi-directional bending in response to varying solvent quality and temperature. We chose ethanol (EtOH) as a cosolvent with water for actuating the bilayer system because pNIPAAm expresses both LCST and UCST phase transitions at particular concentrations of EtOH-water mixtures [49,50]. EtOH also induces a distinct volume collapse in ionized pNaAc gels [51]. As illustrated in Figure 1c, water at temperatures above the LCST causes the pNIPAAm network to dehydrate and shrink, resulting in bending with the pNIPAAm inside (positive curvature). In room temperature EtOH, the pNaAc shrinks more than the pNIPAAm and the bilayer bends with the pNaAc inside (negative curvature). In the next section, we discuss how to tune the bending direction by modulating the solvent and temperature.

To control the bending axis, we utilize an ionoprinting technique to create localized, reversible, crosslinked regions within the pNaAc gel using divalent ions [21]. Briefly, applying an oxidative bias to a flat or patterned metal anode (in this case, Cu) injects ions into the pNaAc as a result of the current through the gel. The Cu^2+^ ions generated at the anode/hydrated gel interface associate with the anionic carboxylic groups on the gel polymer backbone, forming robust ionic crosslinks. A single ionoprinted line across the length of the pNaAc layer results in the generation of in-plane stresses when exposed to appropriate solvents due to the large swelling ratio contrast [45]. The swelling ratio contrast and the resulting stress field leads to inversion of the bending axis of the bilayer sheet (Figure 1d). In water above the LCST, the ionoprint is exposed (*i.e*., on the exterior) and is orthogonal to the bending axis. In room temperature EtOH, the ionoprint is hidden (*i.e*., on the interior) and is parallel to the bending axis. Hence, we present flat bilayer sheets engineered to change both bending direction and axis by simply controlling temperature or solvent quality.

### 3.2. Response Analysis of Individual Gel Layers

**Figure 2:**
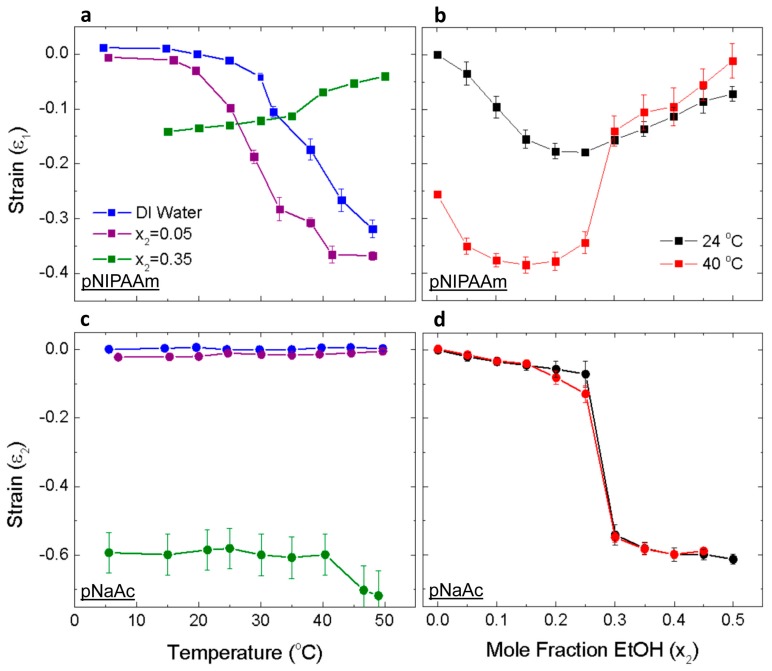
Response of the individual hydrogel layers to temperature and solvent quality. All reported values are relative to the reference state of room temperature water. (**a**) The actuation strain of pNIPAAm gel strips, ε_1_, in various mixtures of EtOH and water as a function of temperature. (**b**) The actuation strain, ε_1_, above and below the LCST as a function of solvent quality. (**c**) The actuation strain of pNaAc gel strips, ε_2_, in various mixtures of EtOH and water as a function of temperature. (**d**) The actuation strain, ε_2_, at 24 °C and 40 °C as a function of solvent quality. The errors bars represent the standard deviation (SD) of five samples.

As a step toward understanding the bilayer bending behavior of the gels, we first characterized the swelling and shrinking behavior of the separate gel networks. Towards this end, we equilibrated gel strips in varying concentrations of EtOH/water with mole fractions *x*_2_ = 0−0.5 and at temperatures below and above the LSCT. Figure 2 shows the strain due to free swelling along the gel length for individual pNIPAAm, ε_1_, and pNaAc, ε_2_, strips. A negative strain implies that the gel shrinks. The length of the gel strips in pure water at 24 °C was used as the reference state. In pure water, the gel begins to shrink sharply above 30 °C, which correlates to the coil-to-globule phase transition incurred by linear chains of pNIPAAm at the LCST (Figure 2a). The pNIPAAm gel shrinks continuously up to 50 °C. Temperatures less than the LCST favor mixing between the polymer and water due to the large enthalpy of mixing incurred by hydrogen bond formation between either the amide groups and water [43,49] carbonyl groups and water [52] or by formation of a hydration shell around the hydrophobic isopropyl group [50]. These bonded water molecules acquire low orientational entropy. Aggregation and phase separation of the pNIPAAm gel at higher temperatures occurs due to the entropy gain in releasing the water molecules.

The LCST shifts to 24 °C by adding a small amount of ethanol, *x*_2_ = 0.05 (Figure 2a). This phenomenon, termed “cononsolvency”, is characterized as the initial decrease in solubility of a polymer by small additions of a favorable cosolvent followed by the expected increase in solubility with the addition of more favorable cosolvent [43,49,50]. Ethanol-water complexes are preferred to NIPAAm-water interactions at certain intermediate concentrations. This is due to the order-making or kosmotrope effect of solutes, such as alcohols or ketones, added to water. The inclusion of a kosmotrope in water leads to the formation of a hydration shell around the solute, which promotes stronger and longer lasting hydrogen bonding than in the bulk [50,53]. Initial increases in the concentration of ethanol decrease the enthalpy of the system and lower the LCST. The decrease of LCST to room temperature at *x*_2_ = 0.05 agrees with the transition observed for linear NIPAAm chains in binary ethanol/water mixtures [49,50]. The kosmotrope effect is limited by the capacity of the water molecules to form hydration shells around the solute. The solute molecules are free to interact with the polymer once an excess of solute is added, which cannot be hydrated by the available water. This will lead to increased solubility of polymer chains, which also results in swelling for a crosslinked polymer. In our case, the pNIPAAm gels swell and exhibit a UCST transition as the EtOH mole fraction exceeds *x*_2_ = 0.25 at 24 °C and *x*_2_ = 0.25 at 40 °C (Figure 2a,b). The decrease in UCST with increasing temperature is most likely due to the destabilization of hydrogen bonds between water, increasing EtOH/pNIPAAm interactions [49].

In pure water and at *x*_2_ = 0.05, the pNaAc gel strip is unresponsive to increasing temperature, up to 50 °C (Figure 2c). This is due to the strong hydrophilic nature of the polymer when its carboxylic groups are ionized (pH > pKa = 4.8) [54]. Upon increasing the EtOH concentration, pNaAc exhibits a sharp, discontinuous volume transition at *x*_2_ = 0.25. The collapse of polyelectrolyte chains in low polarity solvents may be caused by the formation of ion pairs between the fixed ion groups and mobile counter ion [51,55]. This effect becomes more pronounced with decreasing dielectric constant of the external solution and increased ionization of the polyelectrolyte. In the present system, the volume collapse of pNaAc at *x*_2_ = 0.25 is attributed to ion pair formation between COO^−^ and Na^+^ groups, which becomes energetically favorable as compared to the swollen state in a low polarity solvent [56]. Previous experiments of both linear chains [55] and crosslinked pNaAc [51] in binary EtOH mixtures reported chain collapse in the range 0.17 < *x*_2_ < 0.31, which agrees with our data. The effect of increasing EtOH on the chain collapse of pNaAc is not affected by increasing temperature up to 40 °C (Figure 2d). The ability to control the onset and magnitude of the volume swelling response of two individual gel layers enables the design of bilayer actuators that can respond bi-directionally to external stimuli at a specified set of conditions.

### 3.3. Response of Hydrogel Bilayers to External Stimulus

**Figure 3:**
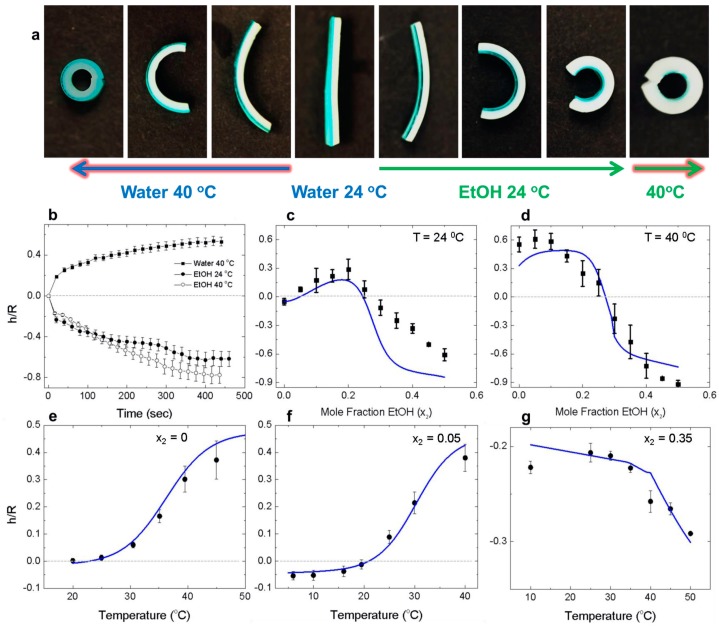
Bending behavior of bilayer gel actuators at various temperatures and EtOH concentrations. (**a**) Photographs of a bilayer gel actuator showing positive curvature in pure water at 40 °C and negative curvature after immersion in EtOH at 24 °C (*x*_2_ = 0.5). The last frame shows the amplification of the bending magnitude as the temperature is increased to 40 °C to induce the UCST behavior of pNIPAAm. The pNaAc layer has been stained with brilliant green dye for visualization. Scale bar = 2 mm. (**b**) The normalized bending curvature as a function of time in pure water and EtOH (*x*_2_ = 0.5). (**c**) Bilayer bending behavior at 24 °C as a function of *x*_2_. (**d**) Bilayer bending behavior at 40 °C as a function of *x*_2_. (**e**) Bilayer bending behavior in pure water as function of *T*. (**f**) Bilayer bending behavior at *x*_2_ = 0.05 as function of *T*. (**g**) Bilayer bending behavior at *x*_2_ = 0.35 as function of *T*. The solid blue lines are the fit curves from Equation (1). The dashed lines provide a guide for the transition from positive to negative curvature.

Next, we characterized the speed and magnitude of the bending response of gel bilayers composed of pNIPAAm and pNaAc. The bilayer system will incur a strain gradient due to the individual responses of the separate layers to the external environment. The increase in elastic energy driven by the strain mismatch is partially alleviated by bending, as shown in Figure 3a. We measured the radius of curvature, *R*, of the bilayers after equilibration in ethanol mixtures, 0 < *x*_2_ < 0.5 and at two temperatures, 24 °C and 40 °C, to determine the bending curvature (1/*R*). The bending curvature was normalized by the total thickness of the bilayer, *h*. The time to reach maximum bending is shown in Figure 3b for bilayers placed in pure water and at *x*_2_ = 0.5. In all cases, the maximal normalized bending curvature, *h*/*R*, was reached after ~6.5 min. After normalizing to the initial thickness (5.2 min/mm), these gel actuators are fast compared to other systems (~400–20 min/mm [10,16,40,41]). Typically, the anisotropic swelling response to external stimuli is due to the combination of a passive layer with an active gel layer. While many other factors determine the swelling response of hydrogels (such as porosity and polymer concentration), we attribute the rapid bending to the combination of two active gel layers.

We used the environment response behavior of the individual gel layers to control the bilayer bending direction. As shown in Figure 3c, the bilayer initially curves towards the pNIPAAm layer (positive values) below the experimentally determined collapse transition point of pNaAc in EtOH (*x*_2_ = 0.25). This is due to the decreasing LCST of the pNIPAAm layer *x*_2_ < 0.05, which enabled the gel to shrink at room temperature. The bilayer attains a flat configuration at *x*_2_ = 0.3, then subsequently exhibits strong bending with pNaAc layer inside (negative curvature). Hence, the bilayer actuator is able to exhibit three programmable states at isothermal conditions. The bending magnitude is greatly increased for the entire solution composition range at 40 °C (Figure 3d). In pure water, the pNIPAAm is well above the LCST (30 °C) and the bilayer bends with strong, positive curvature. With increasing EtOH concentration, the pNaAc layer begins to shrink and forces bending in the negative direction. The bending direction quickly reverses toward the NaAc as the EtOH concentration surpasses *x*_2_ ≈ 0.2. This response due to the complementary effects of pNIPAAm swelling above the UCST, while concurrently the pNaAc shrinks in EtOH. Hence, the UCST behavior provides an amplification effect for negative bending curvature.

The ability to shift the bilayer transition temperature is illustrated in Figure 3e,f. The addition of a small amount of EtOH (*x*_2_ = 0.05) induces positive curvature at 24 °C. In Figure 3g, the bilayer is negatively curved through the whole range of temperatures due to the high EtOH content (*x*_2_ = 0.35). The UCST behavior occurs at 40 °C, amplifying the bending curvature.

A simple model for understanding the directional bending behavior induced by the strain mismatch is provided by Timoshenko’s equation for a bilayer of two linear elastic materials [57,58]. This model has previously been applied to stimuli responsive polymer layers connected to passive gel layers [59] or rigid films [10,60], even though the hydrogels are most likely outside of the linear elastic regime. We apply it here to a bilayer with two actively responsive layers to obtain some qualitative understanding of the bending direction:

(1) $$\frac{h}{R}=\frac{6\left(\varepsilon_{2}-\varepsilon_{1}\right){\left(1+m\right)}^{2}}{\left(3{\left(1+m\right)}^{2}+\left(1+mn\right)\left(m^{2}+\frac{1}{mn}\right)\right)}$$

where *m = a*_1_*/a*_2_ is the ratio of the thicknesses, *n = E*_1_*/E*_2_ is the ratio of the Young’s moduli of the two materials, and ε_1_ and ε_2_ are the actuation strains of the two layers. The subscripts 1 and 2 correspond to the pNIPAAm and pNaAc gels, respectively. For ε_2_ > ε_1_, the deflection of the strip is convex down, indicating positive curvature with the pNIPAAm gel on the inside. The total thickness of the gel bilayers, *h*, was fixed at 1.25 mm (pNaAc = 0.25 mm and pNIPAAm = 1 mm). The Young’s moduli were measured as 227.32 ± 0.079 kPa for the pNaAc gel and 52.91 ± 0.075 kPa for the pNIPAAm gel in room temperature water. The experimental bilayer bending behavior is shown in Figure 3 for the bilayer gels as a function of temperature and solvent quality. The fits from Timoshenko’s equation were obtained utilizing the measured actuation strain values. The modulus values were kept constant, except for the high ethanol case, *x*_2_ = 0.35, without any additional fitting parameters. In all cases the model qualitatively predicted the same directional bending transitions as observed experimentally. In Equation (1), the bending curvature is a function of the ratio of the two moduli. Hence, the model is not significantly affected by changes in the modulus if the relative values remain constant. We observed the best fits at low ethanol concentrations where the pNaAc is unresponsive to temperature. The bulk modulus of pNIPAAm has been shown to increase with increasing temperature as polymer/polymer interactions reinforce the resistance to strain [61,62]. The largest discrepancies between the model and experimental data occur at high concentrations of EtOH. The discrepancies are most likely due to the pNaAc layer incurring large modulus changes during chain collapse and ion pair formation. In order to fit the data in Figure 3g, the modulus value *E*_2_, was set to ~14 GPa. While this value is much too high, the trend of increasing modulus with EtOH concentration is supported by previous work [21]. We demonstrate that Timoshenko’s equation can qualitatively describe the bending behavior of a bilayer gel with two active layers. The quantitative agreement may be improved by measuring the mechanical properties of the individual gel layers as a function of *T* and *x*_2_, which is experimentally challenging and beyond the scope of this paper.

### 3.4. Inversion of Bending Axis by Ionoprinting

**Figure 4:**
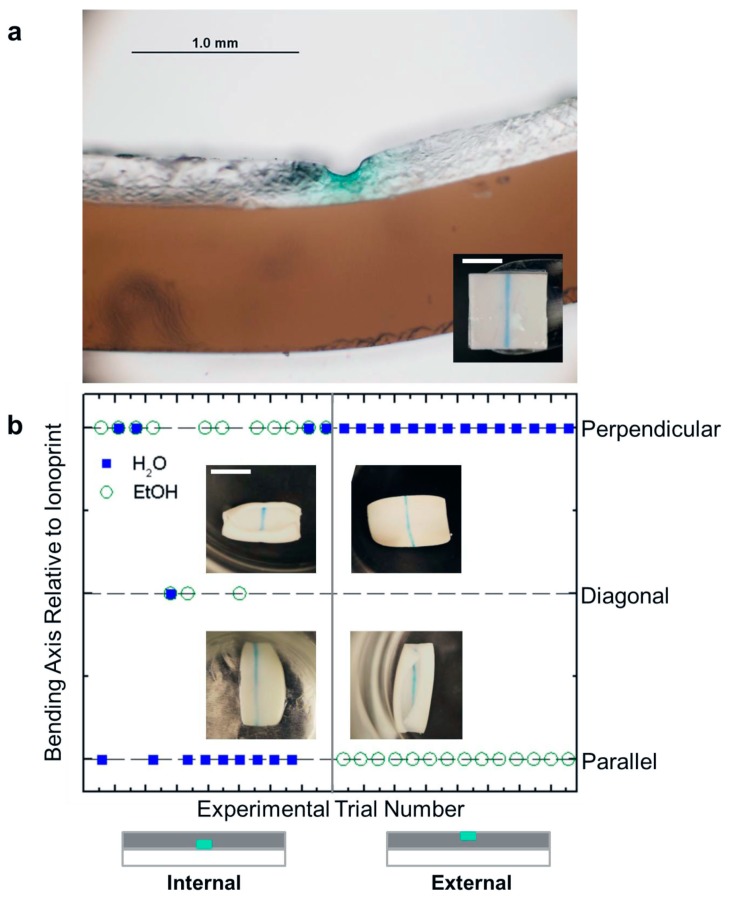
Ionoprinted multi-responsive bilayer actuators. (**a**) Micrograph of the cross-section of the bilayer with an external ionoprint. Inset: Top down view of an ionoprinted bilayer. Scale bar = 12 mm. (**b**) The final orientation of the bending axis relative to the ionoprint direction at 40 °C as a function of experimental trial number. Left column: Gel bilayers with the internal ionoprints located at the interface. Right column: Gel bilayers with the external ionoprints located away from the interface. Insets: Photographs of the bilayer bending behavior. Scale bar = 12 mm.

The ionoprinting technique was used to create robust, ionic crosslinked regions in the pNaAc gel layer to control the bending axis (Figure 4). In pure water, the ionoprints are still able to induce bending after months [21]. We expected the stiffer, crosslinked region to act as a hinge with the ionoprint parallel to the bending axis in both hot water and EtOH. However, we observed serendipitously that the bilayer inverts curvature from parallel to the bending axis in EtOH to orthogonal to the bending axis in hot water for an ionoprint on top (external ionoprint) of the pNaAc layer (Supplementary Material Video S1). The inversion of the bending axis is not fully understood, but there is some precedent in the literature for other bending soft materials. This phenomenon is analogous to simulations and experiments conducted by Nie *et al.* on hydrogels sheets with localized strips of gel with different crosslinking densities [40]. Their finite element model demonstrated the possibility of the hybrid hydrogel to invert its bending axis when the localized strips were crosslinked less than the bulk matrix. Recently, double folding behavior was demonstrated experimentally for PDMS elastomer sheets with localized crosslinked strips of SU-8 photoresist [45]. The sheets initially fold with the strips facing outside and parallel to the bending axis, but immediately reverse and begin to unroll, and then fold with the strips facing inside and orthogonal to the bending axis. The bending behavior is attributed to the bending moments created by the strain difference between the strips and bulk elastomer.

**Figure A1:**
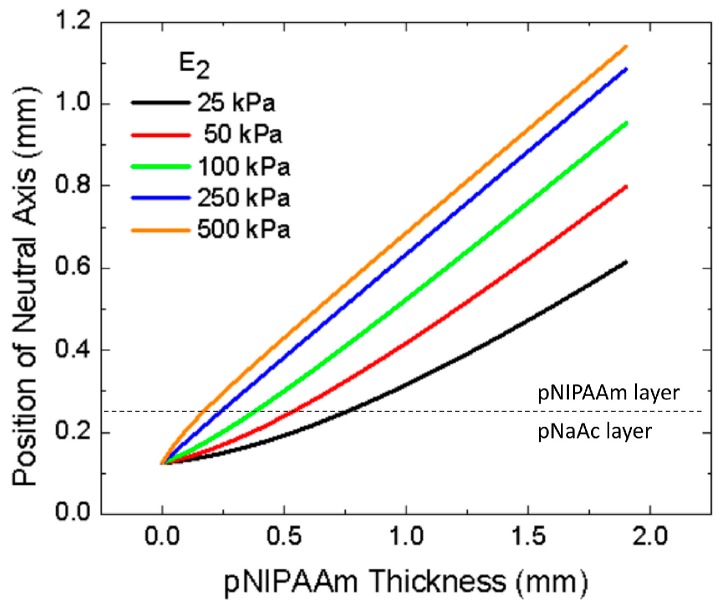
Position of the neutral axis as a function of the pNIPAAm thickness. The dashed line marks the interface between the two gel layers.

Next, we characterized how the location of the ionoprint along the bilayer thickness may affect the rolling behavior. When the ionoprint is located on the bottom of the pNaAc layer (*i.e*., at the interface with the pNIPAAm, which we call an “internal ionoprint”), the bilayer actuators can demonstrate bending behavior directly opposite to the external ionoprint bilayer response (Video S2). Figure 4b shows the final bending axis orientation for the gel actuators relative to the ionoprint direction as a function of experimental trial number. Internal ionoprints result in erratic directionality wherein the ionoprint is diagonal relative to the bending axis. The external ionoprints are more reliable in dictating the bending axis (Figure A1). In summary, the increased crosslink density caused by a single ionoprint can invert the bending axis of the bi-directional bilayers. It is possible to induce the opposite shape conformations by changing the location of the ionoprint within the depth of the bilayer.

Finally, we determined whether the bending behavior observed is a kinetic response or based on the internal stresses developed in solution at equilibrium. Externally ionoprinted bilayers were submerged in 40 °C water for at least 5 min and held by tweezers along the opposing bending axis (orthogonal to the ionoprint). The ionoprinted bilayer immediately snaps to its equilibrium conformation after releasing the tweezers (Video S3).

### 3.5. Demonstration of Complex Shapes using Ionoprinting

**Figure 5:**
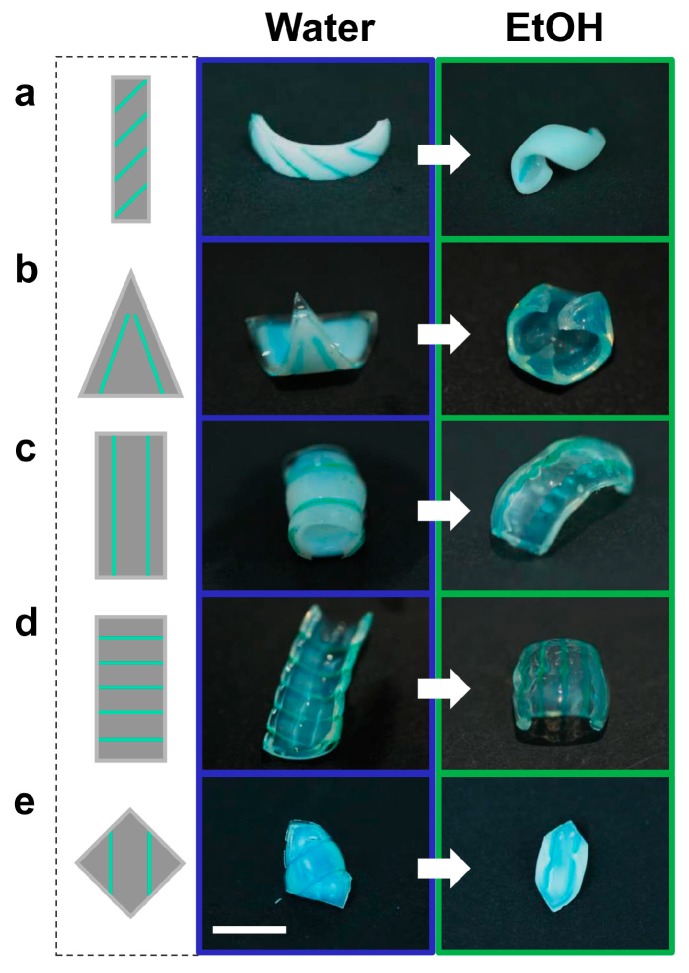
Unique shape responses of hydrogel bilayers enabled by external ionoprints. Left column: Schematic of the ionoprint design Middle column: Equilibrium shape in water at 40 °C. Right column: Equilibrium shape in EtOH at *x*_2_ = 0.5. Scale bar = 5 mm. (**a**–**e**) In pure water above the LCST each actuator exhibits positive curvature and a dominant bending axis orthogonal to the ionoprint direction. In pure EtOH each actuator exhibits negative curvature. The ionoprints resist bending and act as hinges.

The ionoprinting technique enables rapid ionic crosslink patterning initiated by applying voltage to copper wires. By combining it with the multi-responsive bilayers, we can create gel actuator sheets that form unique shapes in response to the external environment. Figure 5 shows various shape transformations for bilayers submerged in pure water at 40 °C and EtOH at *x*_2_ = 0.5. Using only external ionoprints, the bilayers achieve completely different structures and opposite bending directions between the two stimuli. The response time of each of these example actuators occurred within 5 min.

**Figure A2:**
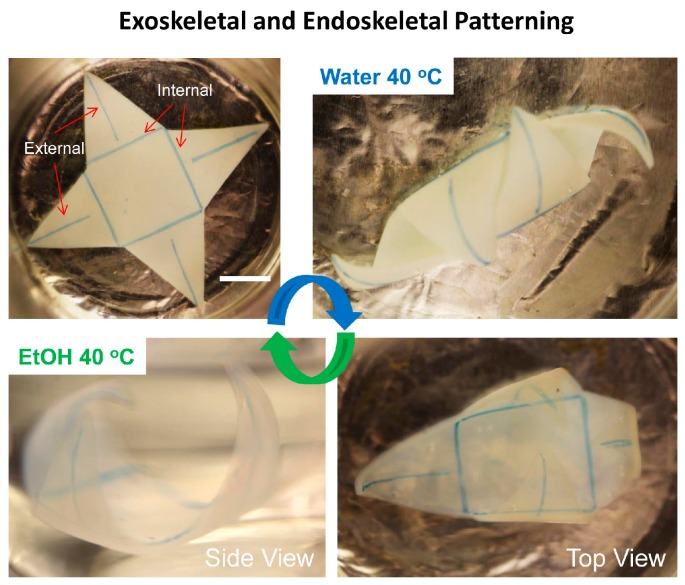
Prototype gel actuator that combines internal and external ionoprints. In water at 40 °C, the actuator resembles a Venus flytrap shape. In EtOH at 40 °C, the actuator resembles a pyramid shape. Scale bar = 1 cm.

The actuators fold relative to the ionoprinted lines according to the simple cases demonstrated with single ionoprints on squares. More complex shapes may be achieved by the combination of both internal and external ionoprinting (see Figure A2). Further insight into the viscoelastic response of the individual gel networks as a function of the external conditions and the relationship on the gel geometry relative to the ionoprints will be necessary in order to gain full control over the final shape transformation.

## 4. Conclusions

We have demonstrated that the combination of multi-responsive hydrogel bilayers with localized crosslinking gradients induced by ionoprinting allows comprehensive control over the bending direction and axis of patterned thin sheets into complex, 3D shapes. The directional bending response can be tuned by modulating the solvent quality and the temperature of the external solution. The ionoprinting regions remain stiff and unresponsive to the external environment. The bulk gel layers undergo differential swelling strains depending on the temperature and solvent composition. The LCST of the pNIPAAm gel can be tuned depending on the EtOH concentration, enabling the bilayers to change direction isothermally. We also demonstrate the use of UCST swelling in the pNIPAAm gel networks, which can produce an amplifying effect during bilayer bending. Ionoprinted lines located at the surface of the pNaAc gel layer reliably and repeatability invert the gel bilayer bending axis between water and EtOH. Opposite conformations relative to the bending axis are possible if the ionoprinted lines are located at the interface between the gel layers. The actuation behavior enabled by these design strategies was used to make gel actuators that transform into pre-designed 3D shapes depending on the environment. Such devices would be useful for applications such as mechanical metamaterials, environment specific encapsulation and chemo-mechanical sensors.

## Supplementary Materials

The following are available online at http://www.mdpi.com/2072-666X/7/6/98/s1. Video S1: External Ionoprint Bilayer Bending. This video shows the externally ionoprinted bilayer response in water (40 °C) and ethanol (40 °C). The video speed was increased by 8× to shorten the viewing time. Video S2: Internal Ionoprint Bilayer Bending. This video shows the internally ionoprinted bilayer response in water (40 °C) and ethanol (40 °C). The video speed was increased by 8× to shorten the viewing time. Video S3: Bilayer Equilibrium Bending Conformation. This video shows an externally ionoprinted bilayers were submerged in 40 °C water and held by tweezers along the opposing bending axis (orthogonal to the ionoprint). The bilayer snaps to its equilibrium conformation after releasing the tweezers. The video speed was increased 4× to shorten the viewing time.

## Appendix A

Overall, the internally ionoprinted bilayers rolled along the opposite bending axis as their external counterparts. The inconsistent bending behavior occurred more often when the bilayers were placed in water at 40 °C. While equilibrating in hot water, the thicker pNIPAAm gel shrinks while the pNaAc thickness practically remains unaffected. This means that the interface between the two gels will approach the middle of the total bilayer thickness. For any bent beam, the bending strain crosses through a plane called the line of neutral strain, or neutral axis, above which the beam is under tension and below which the beam is under compression [60,63]. The final beam length is the same as the original length at the neutral axis, so no stretching or compression due to bending takes place. Bending induced in the bilayer takes place about the neutral axis, which for an isotropic material is located at the geometric centroid. For a composite beam, the neutral axis will depend on the relative cross-sectional area and modulus of the two materials. Note that we do not include the ionoprint for this hypothetical analysis but demonstrate the possibility of a shifting neutral axis where the ionoprint is located. Hence, the stiff, crosslinked region may lose influence over the bending axis when the ionoprint is located at the interface of the bilayer (internal) and passes through the neutral axis.

To support our hypothesis, we calculated the position of the neutral axis, *z*_0_, for the bilayer actuator in pure water at 40 °C. The gel actuator was modeled as a composite beam using Equation (A1):

(A1) $$z_{0}=\frac{E_{1}A_{1}C_{1}+E_{2}A_{2}C_{2}}{E_{1}A_{1}+E_{2}A_{2}}$$

where *A* is cross-sectional area of the gel layer and *C* is the centroid of the layer [63]. As shown in Figure A1, the neutral axis approaches the gel/gel interface as the pNIPAAm layer shrinks in response to temperature. Hence, as the internal ionoprint approaches the line of zero strain, it is possible for it to lose its influence on the final shape conformation. Equation (A1) shows that higher modulus pNIPAAm gels need to be thinner for the line of zero strain to cross the interface. Previous studies have measured that while the pNIPAAm elastic modulus increases overall with temperature, there is a sharp dip that occurs at the LCST. This phenomenon may occur in the bilayer actuators, causing the line of zero strain to cross the interface at higher pNIPAAm thicknesses [62,64]. We made bilayer gels with much thicker pNIPAAm layers (*h*_1_ = 2.5 mm) to determine the final shape conformation when the internal ionoprint remains far from the gel/gel interface. Indeed, we observed that the bending axis is parallel to the ionoprint in pure water at 40 °C and orthogonal to the ionoprint in EtOH *x*_2_ = 0.5 with a success rate of 10/10 trials. However, the bilayers took much longer to respond to the external solutions (~20–30 min) for the thicker gel layers.

We attempted to induce both orthogonal and parallel bending axes in response to the same stimulus by a combination of internal and external ionoprinting (Figure A2). As the pNIPAAm layer shrinks in hot water, the internal ionoprints act as hinges while the gel will bend parallel to the external ionoprints. The competing stresses result in a twisted configuration that resembles a Venus flytrap. In EtOH, we expected the bilayer to remain flat along the external ionoprints as the four arms bend parallel to each ionoprint along the square in order to form a pyramid structure. This is a promising first prototype gel sheet actuator that can adopt multiple complex structures at the macro scale.

## References

[1] Studart A.R., Erb R.M. (2014). Bioinspired materials that self-shape through programmed microstructures. Soft Matter.

[2] Geryak R., Tsukruk V.V. (2014). Reconfigurable and actuating structures from soft materials. Soft Matter.

[3] Khetan S., Burdick J.A. (2010). Patterning hydrogels in three dimensions towards controlling cellular interactions. Soft Matter.

[4] Kuckling D. (2009). Responsive hydrogel layers—From synthesis to applications. Colloid Polym. Sci..

[5] White E.M., Yatvin J., Grubbs J.B., Bilbrey J.A., Locklin J. (2013). Advances in smart materials: Stimuli-responsive hydrogel thin films. J. Polym. Sci. B Polym. Phys..

[6] Asoh T.-A., Kawamura E., Kikuchi A. (2013). Stabilization of electrophoretically adhered gel-interfaces to construct multi-layered hydrogels. RSC Adv..

[7] Morales D., Palleau E., Dickey M.D., Velev O.D. (2013). Electro-actuated Hydrogel Walkers with Dual Responsive Legs. Soft Matter.

[8] Breger J.C., Yoon C.-K., Xiao R., Kwag H.R., Wang M.O., Fisher J.P., Nguyen T.D., Gracias D.H. (2015). Self-Folding Thermo-Magnetically Responsive Soft Microgrippers. ACS Appl. Mater. Interfaces.

[9] Kuckling D., Pareek P. (2008). Bilayer hydrogel assembly. Polymer.

[10] Na J.-H., Evans A.A., Bae J., Chiapelli M.C., Santangelo C.D., Lang R.J., Hull T.C., Hayward R.C. (2015). Programming Reversibly Self-Folding Origami with Micropatterned Photo-Crosslinkable Polymer Trilayers. Adv. Mater..

[11] Nikolov S.V., Yeh P.D., Alexeev A. (2014). Self-Propelled Microswimmer Actuated by Stimuli-Sensitive Bilayered Hydrogel. ACS Macro Lett..

[12] Stoychev G., Puretskiy N., Ionov L. (2011). Self-folding all-polymer thermoresponsive microcapsules. Soft Matter.

[13] Xu B., Jiang H., Li H., Zhang G., Zhang Q. (2015). High strength nanocomposite hydrogel bilayer with bidirectional bending and shape switching behaviors for soft actuators. RSC Adv..

[14] Zakharchenko S., Puretskiy N., Stoychev G., Stamm M., Ionov L. (2010). Temperature controlled encapsulation and release using partially biodegradable thermo-magneto-sensitive self-rolling tubes. Soft Matter.

[15] Zheng W.J., An N., Yang J., Zhou J., Chen Y.M. (2015). Tough Al-alginate/poly(*N*-isopropylacrylamide) Hydrogel with Tunable LCST for Soft Robotics. ACS Appl. Mater. Interfaces.

[16] Bassik N., Abebe B.T., Laflin K.E., Gracias D.H. (2010). Photolithographically patterned smart hydrogel based bilayer actuators. Polymer.

[17] Hauser A.W., Evans A.A., Na J.-H., Hayward R.C. (2015). Photothermally Reprogrammable Buckling of Nanocomposite Gel Sheets. Angew. Chem. Int. Ed..

[18] Thérien-Aubin H., Wu Z.L., Nie Z., Kumacheva E. (2013). Multiple Shape Transformations of Composite Hydrogel Sheets. J. Am. Chem. Soc..

[19] Yao C., Liu Z., Yang C., Wang W., Ju X.-J., Xie R., Chu L.-Y. (2015). Poly(*N*-isopropylacrylamide)-Clay Nanocomposite Hydrogels with Responsive Bending Property as Temperature-Controlled Manipulators. Adv. Funct. Mater..

[20] Zhang X., Pint C.L., Lee M.H., Schubert B.E., Jamshidi A., Takei K., Ko H., Gillies A., Bardhan R., Urban J.J. (2011). Optically- and Thermally-Responsive Programmable Materials Based on Carbon Nanotube-Hydrogel Polymer Composites. Nano Lett..

[21] Palleau E., Morales D., Dickey M.D., Velev O.D. (2013). Reversible patterning and actuation of hydrogels by electrically assisted ionoprinting. Nat. Commun..

[22] Bae J., Na J.-H., Santangelo C.D., Hayward R.C. (2014). Edge-defined metric buckling of temperature-responsive hydrogel ribbons and rings. Polymer.

[23] Kim J., Hanna J.A., Byun M., Santangelo C.D., Hayward R.C. (2012). Designing Responsive Buckled Surfaces by Halftone Gel Lithography. Science.

[24] Kim J., Hanna J.A., Hayward R.C., Santangelo C.D. (2012). Thermally responsive rolling of thin gel strips with discrete variations in swelling. Soft Matter.

[25] Lee B.P., Konst S. (2014). Novel Hydrogel Actuator Inspired by Reversible Mussel Adhesive Protein Chemistry. Adv. Mater..

[26] Ahadian S., Ramón-Azcón J., Estili M., Liang X., Ostrovidov S., Shiku H., Ramalingam M., Nakajima K., Sakka Y., Bae H. (2014). Hybrid hydrogels containing vertically aligned carbon nanotubes with anisotropic electrical conductivity for muscle myofiber fabrication. Sci. Rep..

[27] Wu Z.L., Moshe M., Greener J., Therien-Aubin H., Nie Z., Sharon E., Kumacheca E. (2013). Three-dimensional shape transformations of hydrogel sheets induced by small-scale modulation of internal stresses. Nat. Commun..

[28] Peppas N.A., Hilt J.Z., Khademhosseini A., Langer R. (2006). Hydrogels in Biology and Medicine: From Molecular Principles to Bionanotechnology. Adv. Mater..

[29] Slaughter B.V., Khurshid S.S., Fisher O.Z., Khademhosseini A., Peppas N.A. (2009). Hydrogels in Regenerative Medicine. Adv. Mater..

[30] Kwon G.H., Park J.Y., Kim J.Y., Frisk M.L., Beebe D.J., Lee S.-H. (2008). Biomimetic Soft Multifunctional Miniature Aquabots. Small.

[31] Maeda S., Hara Y., Sakai T., Yoshida R., Hashimoto S. (2007). Self-Walking Gel. Adv. Mater..

[32] Murdan S. (2003). Electro-responsive drug delivery from hydrogels. J. Control. Release.

[33] Peppas N.A., Galaev I., Mattiasson B. (2007). Drug Delivery Using Smart Polymers: Recent Advances. Smart Polymers: Applications in Biotechnology and Biomedicine.

[34] Beebe D.J., Moore J.S., Bauer J.M., Yu Q., Liu R.H., Devadoss C., Jo B.-H. (2000). Functional hydrogel structures for autonomous flow control inside microfluidic channels. Nature.

[35] Lin I.-H., Birchall L.S., Hodson N., Ulijn R.V., Webb S.J. (2013). Interfacing biodegradable molecular hydrogels with liquid crystals. Soft Matter.

[36] Jin L., Zhao Y., Liu X., Wang Y., Ye B., Xie Z., Gu Z. (2012). Dual signal glucose reporter based on inverse opal conducting hydrogel films. Soft Matter.

[37] Ionov L. (2014). Hydrogel-based actuators: Possibilities and limitations. Mater. Today.

[38] O’Grady M.L., Kuo P., Parker K.K. (2009). Optimization of Electroactive Hydrogel Actuators. ACS Appl. Mater. Interfaces.

[39] Sun J.-Y., Zhao X., Illeperuma W.R.K., Chaudhiri O., Oh K.H., Mooney D.J., Vlassak J.J., Suo Z. (2012). Highly stretchable and tough hydrogels. Nature.

[40] Wei Z., Jia Z., Athas J., Wang C., Raghavan S.R., Li T., Nie Z. (2014). Hybrid hydrogel sheets that undergo pre-programmed shape transformations. Soft Matter.

[41] Thérien-Aubin H., Moshe M., Sharon E., Kumacheva E. (2015). Shape transformations of soft matter governed by bi-axial stresses. Soft Matter.

[42] Ma C., Li T., Zhao Q., Yang X., Wu J., Luo Y., Xie T. (2014). Supramolecular Lego Assembly towards Three-Dimensional Multi-Responsive Hydrogels. Adv. Mater..

[43] Schild H.G., Muthukumar M., Tirrell D.A. (1991). Cononsolvency in mixed aqueous solutions of poly(*N*-isopropylacrylamide). Macromolecules.

[44] Yu C., Duan Z., Yuan P., Li Y., Su Y., Zhang X., Pan Y., Dai L.L., Nuzzo R.G., Huang Y. (2012). Electronically Programmable, Reversible Shape Change in Two- and Three-Dimensional Hydrogel Structures. Adv. Mater..

[45] Motala M.J., Perlitz D., Daly C.M., Yuan P., Nuzzo R.G., Hsia K.J. (2015). Programming matter through strain. Extreme Mech. Lett..

[46] Asoh T.-A., Kikuchi A. (2010). Electrophoretic adhesion of stimuli-responsive hydrogels. Chem. Commun..

[47] American Society for Testing Materials (2010). Test Method for Tensile Properties of Plastics.

[48] Schneider C.A., Rasband W.S., Eliceiri K.W. (2012). NIH Image to ImageJ: 25 years of image analysis. Nat. Meth..

[49] Costa R.O., Freitas R.F. (2002). Phase behavior of poly(*N*-isopropylacrylamide) in binary aqueous solutions. Polymer.

[50] Bischofberger I., Calzolari D.C.E., De Los Rios P., Jelezarov I., Trappe V. (2014). Hydrophobic hydration of poly-N-isopropyl acrylamide: A matter of the mean energetic state of water. Sci. Rep..

[51] Nishiyama Y., Satoh M. (2000). Swelling behavior of poly(acrylic acid) gels in aqueous ethanol—Effects of counterion species and ionic strength. Macromol. Rapid Commun..

[52] Lee S.G., Pascal T.D., Koh W., Brunello G.F., Goddardlll W.A., Jang S.S. (2012). Deswelling Mechanisms of Surface-Grafted Poly(NIPAAm) Brush: Molecular Dynamics Simulation Approach. J. Phys. Chem. C.

[53] Raschke T.M., Levitt M. (2005). Nonpolar solutes enhance water structure within hydration shells while reducing interactions between them. PNAS.

[54] Baker B.A., Murff R.L., Milam V.T. (2010). Tailoring the mechanical properties of polyacrylamide-based hydrogels. Polymer.

[55] Srikant S., Muralidharan S.S., Natarajan U. (2012). Behaviour of hydrogen bonding and structure of poly(acrylic acid) in water–ethanol solution investigated by explicit ion molecular dynamics simulations. Mol. Simul..

[56] Khokhlov A.R., Kramarenko E.Y. (1996). Weakly Charged Polyelectrolytes: Collapse Induced by Extra Ionization. Macromolecules.

[57] Timoshenko S. (1925). Analysis of Bi-metal Thermostats. J. Opt. Soc. Am..

[58] Freund L.B., Floro J.A., Chason E. (1999). Extensions of the Stoney formula for substrate curvature to configurations with thin substrates or large deformations. Appl. Phys. Lett..

[59] Guo W., Li M., Zhou J. (2013). Modeling programmable deformation of self-folding all-polymer structures with temperature-sensitive hydrogels. Smart Mater. Struct..

[60] Christophersen M., Shapiro B., Smela E. (2006). Characterization and modeling of PPy bilayer microactuators: Part 1. Curvature. Sens. Actuators B Chem..

[61] Schmidt S., Zeiser M., Hellweg T., Duschl C., Fery A., Mohwald H. (2010). Adhesion and Mechanical Properties of PNIPAM Microgel Films and Their Potential Use as Switchable Cell Culture Substrates. Adv. Funct. Mater..

[62] Hirotsu S. (1990). Elastic anomaly near the critical point of volume phase transition in polymer gels. Macromolecules.

[63] Ferdinand P.B., Johnston E.R., DeWolf J.T., Mazurek D.F. (2012). Statics and Mechanics of Materials.

[64] Voudouris P., Florea D., van der Schoot P., Wyss H.M. (2013). Micromechanics of temperature sensitive microgels: Dip in the Poisson ratio near the LCST. Soft Matter.

